# Draf III Procedure Using Multiple Pedicled Mucosal Flaps for Refractory Frontal Sinusitis: A Case Report

**DOI:** 10.7759/cureus.101841

**Published:** 2026-01-19

**Authors:** Naoki Ashida, Miyu Mori, Takeshi Tsuda, Kanako Akita, Hidenori Inohara

**Affiliations:** 1 Department of Otolaryngology - Head and Neck Surgery, Osaka University Graduate School of Medicine, Suita, JPN; 2 Department of Otolaryngology - Head and Neck Surgery, Osaka General Medical Center, Osaka, JPN

**Keywords:** draf iii procedure, endoscopic modified lothrop procedure, endoscopic sinus surgery, pedicled mucosal flap, refractory frontal sinusitis

## Abstract

The Draf III procedure may serve as a surgical option for patients with frontal sinusitis that is refractory to standard treatment or prone to recurrence. However, postoperative restenosis caused by neo-osteogenesis and granulation tissue formation remains a major clinical problem. Coverage of exposed bone using mucosal grafts or pedicled flaps has been advocated to prevent restenosis, but adequate flap reconstruction may be difficult in revision cases with marked bony overgrowth. We report a case of a 62-year-old woman with refractory frontal sinusitis associated with IgG4-related disease and extensive neo-osteogenesis. The patient had undergone multiple prior endoscopic sinus surgeries, resulting in the loss of normal anatomical landmarks and progressive frontal sinus obstruction despite medical therapy, including biological therapy. A Draf III procedure was then performed. Given the extensive bone exposure and anatomical limitations, reconstruction was achieved using a combination of multiple mucosal flaps, including a superior lateral anterior pedicle flap, a septoturbinal flap, a Hadad-Bassagasteguy flap, and free mucosal grafts. Postoperatively, frontal sinus patency was maintained, and the patient’s frontal headache resolved without the need for analgesics at the six-month follow-up. In refractory frontal sinusitis with marked bony overgrowth and a high risk of restenosis, particularly in revision cases, reconstruction using a combination of multiple mucosal flaps tailored to the remaining anatomy may be crucial for maintaining frontal sinus patency.

## Introduction

The Draf III procedure is effective for refractory and recurrent frontal sinusitis. However, postoperative restenosis caused by neo-osteogenesis and granulation tissue formation may complicate management and adversely affect outcomes. Previous studies have reported increased revision rates among patients with certain comorbidities, including aspirin-exacerbated respiratory disease, allergic rhinitis, and bronchial asthma. In addition, inadequate bony removal during surgery, reflected by insufficient intraoperative neo-ostium size, has been shown to correlate strongly with postoperative restenosis and surgical failure [1,2]. To prevent restenosis, coverage of the newly created frontal neo-ostium with either free mucosal grafts or pedicled flaps is important. Pedicled flaps have several advantages, including rapid epithelialization, reduced scar formation, and lower infection rates [3]. However, in revision cases with the loss of normal anatomy or in cases with marked neo-osteogenesis requiring extensive drilling, adequate elevation and coverage with a single pedicled flap may be difficult.

We report a case of recurrent refractory frontal sinusitis with marked neo-osteogenesis treated with the Draf III procedure, in which favorable postoperative outcomes were achieved using a combination of a superior lateral anterior pedicle (SLAP) flap [4], a septoturbinal flap (STF) [5], a Hadad-Bassagasteguy (H-B) flap [6], and free mucosal grafts.

## Case presentation

The patient was a 62-year-old woman with a history of bronchial asthma. Eleven years prior, she underwent endoscopic sinus surgery (ESS) and septoplasty at another institution for chronic rhinosinusitis (CRS) with olfactory disturbance and nasal obstruction, and histopathology confirmed IgG4-related disease. Her sinonasal symptoms recurred shortly after surgery and remained refractory to medical management. She later developed steroid-refractory asthma with pulmonary nodules and was diagnosed with eosinophilic granulomatosis with polyangiitis, which was managed with biologic therapies, including rituximab and later mepolizumab. Six years after the initial surgery, she was referred to our departments of internal medicine and otolaryngology for further evaluation and treatment.

At the initial visit, she complained of a severe headache in addition to olfactory disturbance and nasal obstruction. Nasal endoscopy revealed residual leftward septal deviation and bilateral nasal polyps. Paranasal sinus CT revealed residual septations in the bilateral ethmoid sinuses and diffuse soft tissue opacification involving the bilateral paranasal sinuses, and recurrent CRS was diagnosed. Revision surgery was planned, and repeat ESS and septoplasty were performed. Although attempts were made to remove residual septations and nasal polyps, marked mucosal inflammation and bony sclerosis around the frontal sinuses limited adequate frontal sinus ventilation, resulting in persistent bilateral frontal sinusitis. Postoperatively, her headache, olfactory disturbance, and nasal obstruction improved transiently; however, nasal polyps and symptoms recurred soon after. In the subsequent year, elevated fractional exhaled nitric oxide levels were noted, reflecting persistent systemic eosinophilic inflammation, and the biological therapy was changed from mepolizumab to benralizumab. Four years prior, treatment was switched to dupilumab, which led to marked improvement in olfactory dysfunction and nasal obstruction related to ethmoid sinus disease. However, the severe headache caused by the frontal sinusitis persisted, necessitating the regular use of multiple analgesics; therefore, further surgical intervention was planned.

**Figure 1:**
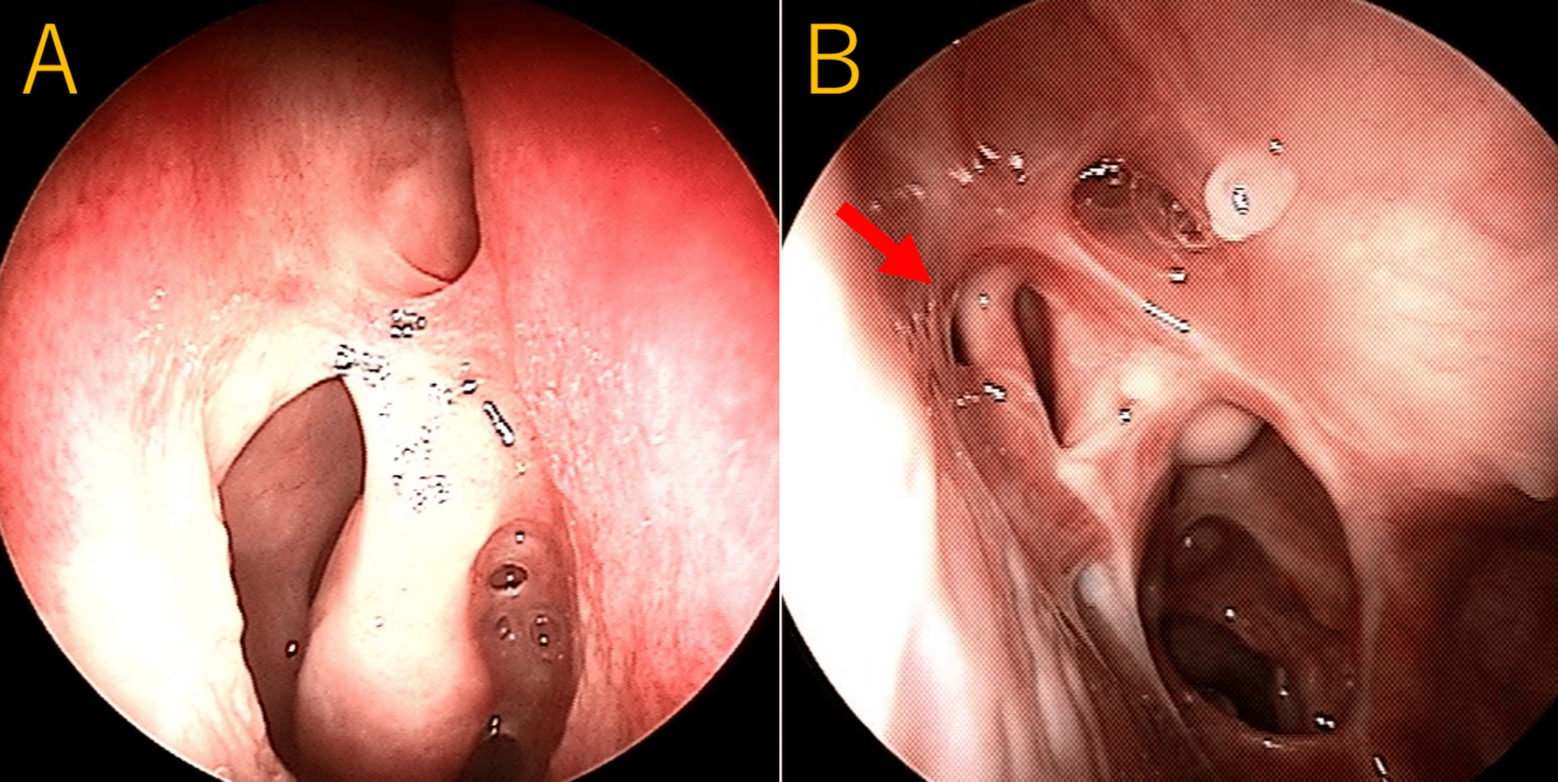
Preoperative endoscopic findings of the nasal cavity (A) Right side: extensive nasal mucosa adhesions are detected. (B) Left side: the middle turbinate has been resected. A septal perforation (a red arrow) with mucosal adhesions is detected in the superior portion of the nasal septum.

Preoperative nasal endoscopy showed extensive scarring and adhesions in both nasal cavities, with prior resection of the left middle turbinate (Figure 1A, 1B).

**Figure 2:**
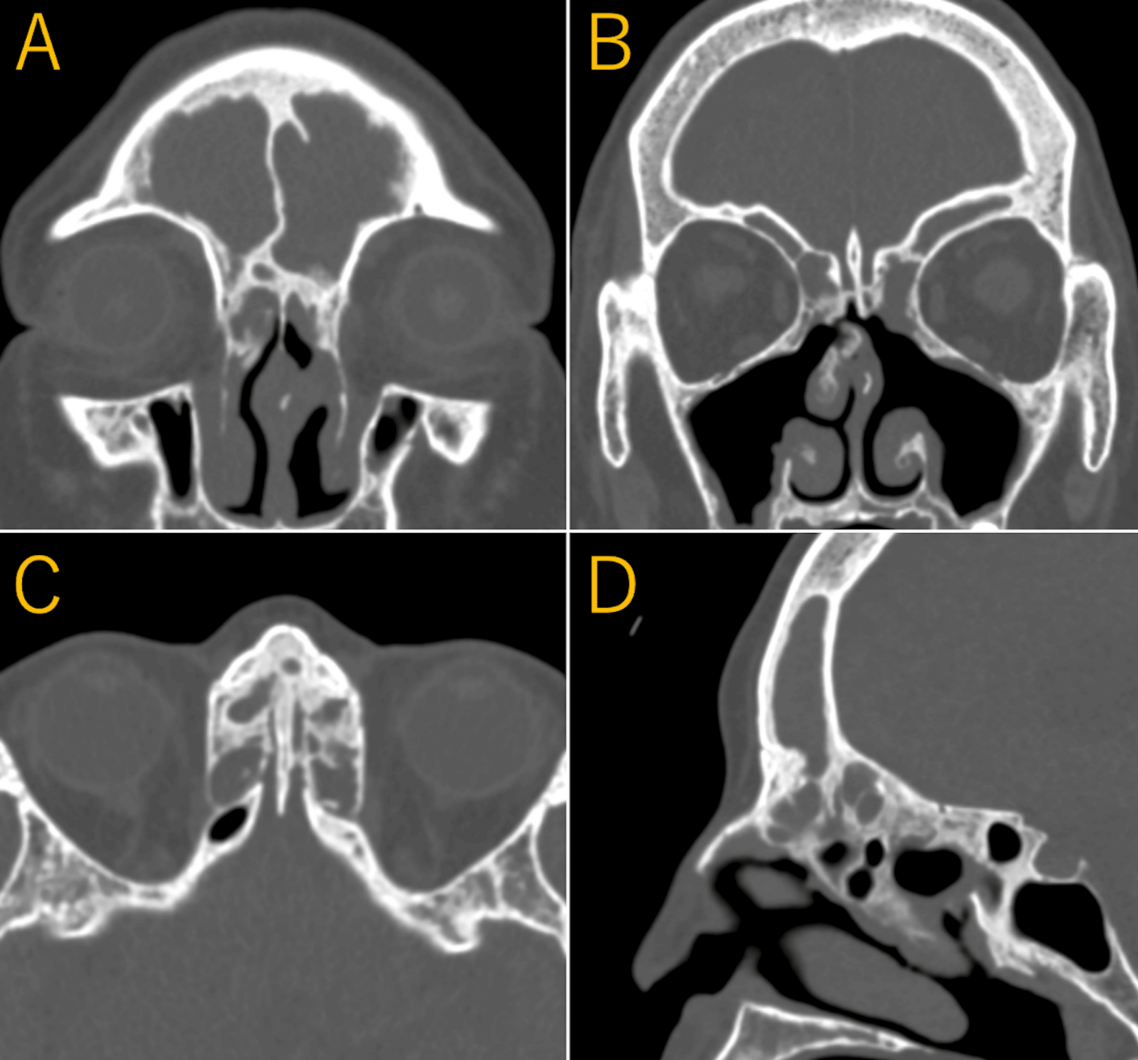
Preoperative CT findings (A, B) Marked neo-osteogenesis around the frontal sinus drainage pathway with frontal sinus opacification. A septal perforation is present in the superior nasal septum. (C) Axial CT image shows severe osteoneogenesis involving the ethmoid sinus. (D) Sagittal CT image (right side) demonstrates extensive osteoneogenesis extending from the frontal sinus pathway to the anterior ethmoid region.

Paranasal sinus CT revealed marked bony proliferation extending from the frontal sinus to the ethmoid sinus, with significant narrowing of the frontal sinuses and complete soft tissue opacification. In addition, a perforation was identified in the superior portion of the nasal septum (Figure 2A-2D).

**Figure 3:**
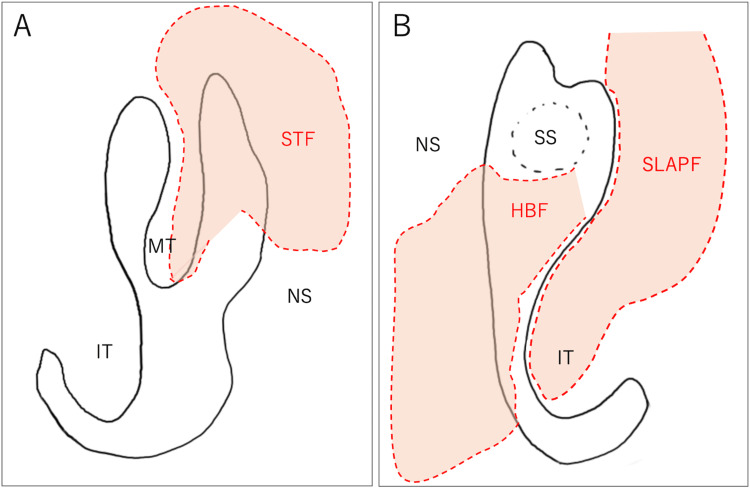
Schematic illustration of mucosal incisions and flap elevation (A) Right nasal cavity. (B) Left nasal cavity. Dashed lines indicate the mucosal incision lines. The shaded areas represent the elevated mucosal flaps. HBF, Hadad-Bassagasteguy flap; IT, inferior turbinate; MT, middle turbinate; NS, nasal septum; SLAPF, superior lateral anterior pedicle flap; SS, sphenoid sinus; STF, septoturbinal flap Image created by Naoki Ashida

Based on these findings, refractory recurrent frontal sinusitis was diagnosed, and a Draf III procedure under general anesthesia was performed. Given the significant bone proliferation and due to the past operation, the plan included creating multiple mucosal pedicles and free flaps before the Draf procedure to prevent postoperative restenosis. A SLAP flap was created in the left nasal cavity using the lateral wall and mucosa, and the inferior turbinate mucosa supplied by the facial artery. Although an attempt was made to create an SLAP flap on the right side, the mucosa was too fragile; therefore, a free mucosal graft was harvested from the inferior turbinate. An STF using the septal mucosa supplied by the anterior ethmoidal artery of the middle turbinate lateral wall was created in the right nasal cavity. In the left nasal cavity, an H-B flap using the septal mucosa supplied by the sphenopalatine artery was elevated. Adhesions were present along the nasal septum due to prior surgery; however, careful sharp dissection allowed safe elevation of both flaps without enlargement of the septal perforation. The schema indicating the incision lines and flaps is shown in Figure 3A, 3B.

**Figure 4:**
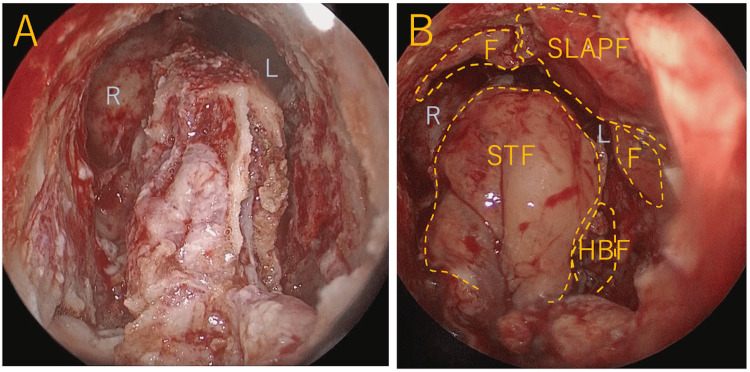
Intraoperative endoscopic findings (A) After extensive bone drilling during the Draf III procedure. (B) Coverage of the exposed bone using pedicled mucosal flaps and a free mucosal graft. F, free mucosal graft; HBF, Hadad-Bassagasteguy flap; L, left frontal sinus; R, right frontal sinus; SLAPF, superior lateral anterior pedicle flap; STF, septoturbinal flap

Next, a standard Draf type III procedure was performed. Dissection on both sides was extended to the first olfactory filament, and bone drilling of the frontal sinus floor was performed. Drilling was performed laterally until the lacrimal sac and anteriorly until the subcutaneous tissue was reached. There was also significant bone proliferation from the posterior wall of the frontal sinus to the ethmoid sinus, and extensive bone drilling was required to widely open the frontal sinus. Copious pus was found in the frontal sinus and was irrigated with saline solution. The left SLAP flap and free mucosa were used to cover the anterior bone-exposed area. The STF and H-B flap were used to cover the skull base side of the bone-exposed area, and the surgery was completed (Figure 4A, 4B).

**Figure 5:**
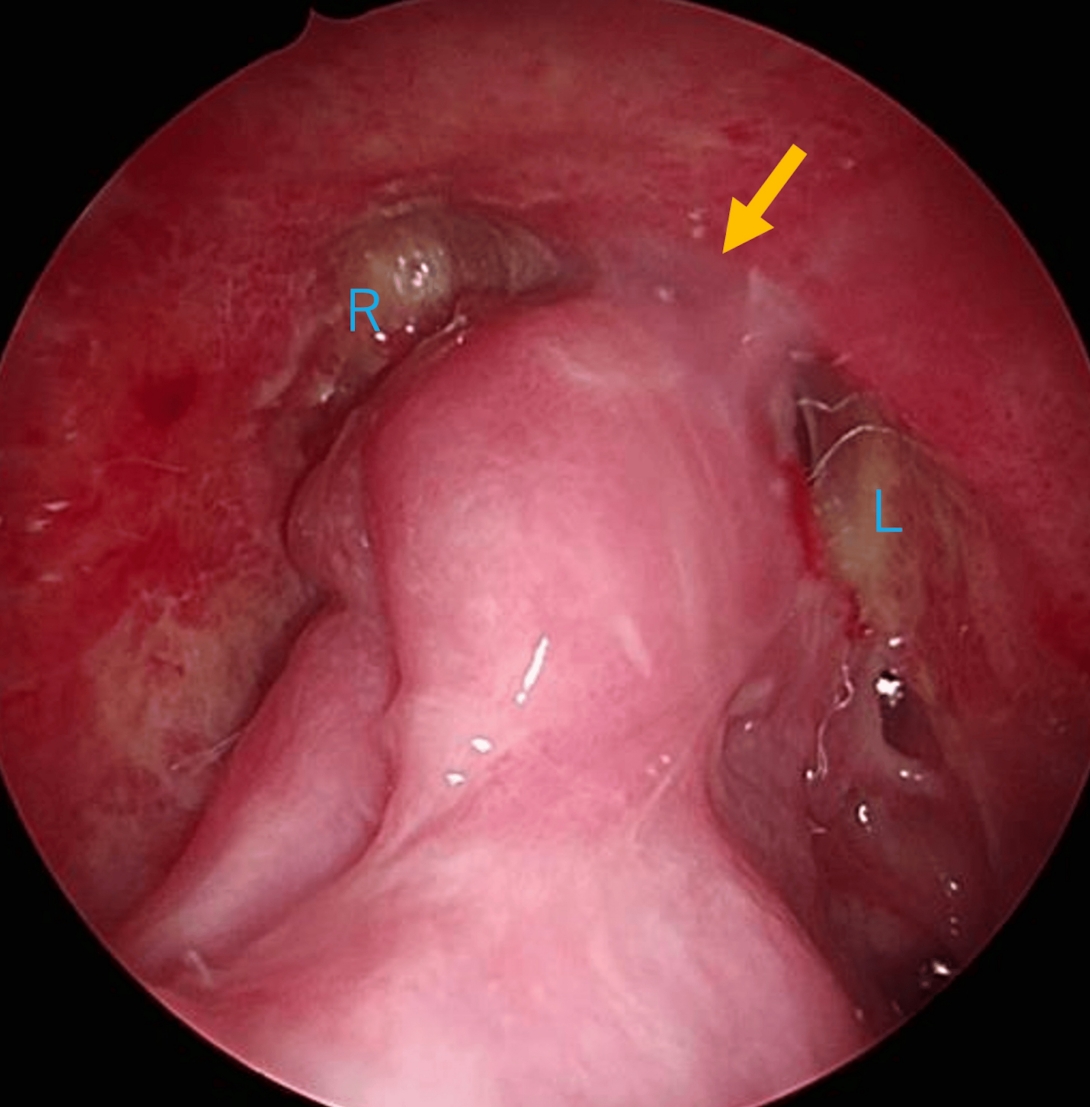
Postoperative endoscopic findings at six months Endoscopic examination performed six months postoperatively shows mild narrowing and partial adhesions in both frontal sinuses, with preservation of sinus patency. A yellow arrow highlights a mild adhesion. L, left frontal sinus; R, right frontal sinus

Postoperatively, the patient continued her preexisting medical therapy, including regular nasal saline irrigation, intranasal corticosteroid spray, and dupilumab. The patient’s headache gradually improved postoperatively and resolved within one month, with the discontinuation of analgesic use. Nasal endoscopy performed six months postoperatively revealed mild narrowing and partial adhesions in both frontal sinuses, although patency was preserved (Figure 5).

## Discussion

The Draf III procedure is a useful surgical technique for the salvage treatment of refractory and recurrent bilateral frontal sinusitis. However, postoperative restenosis due to bone regeneration or granulation tissue formation continues to be a common clinical problem. Recent meta-analyses have shown that symptom improvement in CRS was 82.3%, but restenosis occurred in 17.1% of cases, and 9% of those required reoperation [7]. Inadequate bony removal has been identified as a factor leading to unsuccessful Draf III procedures, with the intraoperative size of the neo-ostium being strongly correlated with postoperative patency [7-9]. Another important factor influencing failure is the exposure of bone surfaces due to drilling. Osteitis occurring on exposed bone surfaces induces fibrosis and bone regeneration, which can lead to restenosis [10]. Therefore, it is important to cover the exposed bone with either free mucosa or a pedicled mucosal flap. Multiple studies have reported a significant reduction in restenosis rates when the exposed area is covered with free or pedicled mucosal flaps [11-13]. While satisfactory outcomes have been reported with free mucosal grafts, pedicled mucosal flaps have been shown to be superior, providing faster epithelialization, reduced scar formation and infection rates, and better preservation of postoperative physiological function [3,14].

Various types of pedicled flaps have been reported for use in Draf III procedures. Among them, the SLAP flap was described by Omura et al. in 2018 as a pedicled flap harvested from the lateral nasal wall, including the inferior turbinate mucosa, and supplied by branches of the facial artery [4]. This flap allows the harvest of a large fresh nasal mucosa sample with a simple procedure that does not obstruct the surgical field. In the present case, the SLAP flap was extremely useful for covering an extensive defect of the anterior wall of the frontal sinus. In the standard Draf III procedure, extensive bone removal from the posterior wall is not typically required. However, in cases with marked neo-osteogenesis, as in the present case, additional thinning or removal of the posterior wall bone may be necessary to ensure long-term patency. The STF, which uses the lateral mucosa of the nasal septum and middle turbinate supplied by the anterior ethmoidal artery [5,15], and the septal mucosal flap (SMF) based on the anterior ethmoidal artery have been reported to cover the posterior wall of the frontal sinus [16].

In the present case, reconstruction using the STF was performed on the right side. On the left side, previous middle turbinate resection and upper septal perforation limited the applicability of an STF or an SMF; therefore, we used an H-B flap, which is commonly used in skull base surgery [6]. Although the use of the H-B flap for Draf procedures in refractory frontal sinusitis has not been previously described, it represents a useful alternative when an STF or an SMF is unavailable because of prior surgery.

In addition to local surgical factors, underlying type 2 inflammatory conditions may also influence postoperative wound healing and the risk of restenosis. Previous studies have reported higher revision rates and poorer surgical outcomes in patients with CRS associated with asthma and nasal polyps, conditions characterized by persistent type 2 inflammation [1,17]. Such inflammatory endotypes have been linked to enhanced tissue edema, fibrosis, and osteitis, which may predispose patients to impaired mucosal regeneration and increased neo-osteogenesis following extensive drilling. In the present case, the patient had asthma-associated CRS with nasal polyps, suggesting a type 2 inflammatory background that may have increased the risk of postoperative restenosis.

In this context, complete bony removal combined with meticulous coverage of exposed bone using multiple pedicled mucosal flaps likely played a critical role in promoting epithelialization and maintaining frontal sinus patency during the postoperative follow-up period. For six months postoperatively, the patient’s symptoms were well-controlled. In Draf III procedures for refractory frontal sinusitis with extensive new bone exposure and a high risk of restenosis, as in this case, reconstruction combining multiple flaps according to anatomical constraints is important. Reports indicate that the neo-ostium may gradually stenose over two years postoperatively [18], suggesting that long-term follow-up is also required in this case.

## Conclusions

In refractory frontal sinusitis with marked bony overgrowth, extensive bone removal often results in wide areas of exposed bone, which may compromise surgical outcomes. Adequate mucosal coverage is therefore essential. In revision surgery, loss of normal anatomical structures often limits the creation of conventional mucosal flaps. In such cases, reconstruction using multiple flaps derived from the remaining tissue may be necessary.
